# Application of Henna as a Durable Skin Marker in Orthopedic Surgery

**DOI:** 10.7759/cureus.10631

**Published:** 2020-09-24

**Authors:** Mukul Mohindra, Amit Meena

**Affiliations:** 1 Orthopaedics, Central Institute of Orthopaedics, Vardhman Mahavir Medical College and Safdarjung Hospital, New Delhi, IND

**Keywords:** henna, mehndi, pre-operative marker

## Abstract

Background

Many orthopedic procedures require skin marking well before surgery. Marks made with most conventional marking pens fade with vigorous skin preparation required before orthopedic procedures, whereas marks with henna may safely last almost up to three weeks. Thereby, we intend to describe applications of henna, a readily available, cost-effective material, as a durable skin marker for various indications in orthopedics.

Methods

A total of 120 patients with varied indications for skin marking were randomized into two equal-sized groups of 60 each. Group A patients were marked with henna and group B patients with a permanent skin marker before the intended surgery. All patients had a routine pre-operative bath one day before surgery and vigorous skin preparation using chlorhexidine and ethanol-based scrubs just before surgery in the operating room. The two markers were compared for the following variables: photographic record to determine fading of the mark after scrubbing, in terms of successful execution of the procedure, patients’ acceptability of the mark, any allergy or infection, and final time of disappearance of the mark.

Results

Marking with henna was clearly visible during all but one surgical procedure even after vigorous pre-operative skin preparation, thereby ensuring minimal use of fluoroscopy in henna-marked patients. The fading of the mark and use of fluoroscopy was significantly low in group A (p<0.05). There was no complication associated with marking with either marking method. Marks with henna disappeared on an average nine days later than with permanent marker. In terms of acceptability of mark, henna was preferred by almost all patients.

Conclusions

Henna paste is an ideal substance for use as a skin marker in conditions, such as foreign body removal, vertebral level identification, nail dynamization, marking tibial physis in children, and sequestrum in non-discharging osteomyelitis, and in marking blood vessel course in tumor surgery or in volar ganglion removal. Pre-operative skin marking with henna ensures minimal use of fluoroscopy, and it is particularly efficacious when ultrasound is used for localization as it can be directly applied over gel film.

## Introduction

All orthopedic procedures must be precisely targeted not only to be efficacious and effective but also to avoid inadvertent damage to normal healthy tissue. Attempting to avoid this deviation in the target point, we often end up making indiscriminate use of intra-operative fluoroscopy that incurs harm to both the patient and the surgeons. To overcome this problem, we need skin marking pre-operatively so that the exact targeted area can be identified precisely. Skin marking with a permanent marker has been used since long just before the surgery, but marks made with most conventional marking pens fade or disappear with vigorous skin preparation that is generally required before performing orthopedic procedures. Permanent skin marker pen is associated with the high cost if it is “for single use”, and if it is reusable, then the risk of cross-infection and sterility of the pen should be kept in mind. Moreover, many patients refuse permanent tattoo dots on cosmetic or religious grounds or because they simply do not wish to be reminded forever about the ailment. Thereby, we intend to describe the use of henna, a low cost widely available material, as a durable pre-operative skin marker in orthopedic procedures. The use of henna has already been described in surgery [1-3] and radiotherapy [2]. To the best of our knowledge, no report in the literature exists that describes the applications of henna in various orthopedic procedures, and thereby we wish to discuss the same in this article.

## Materials and methods

**Figure 1:**
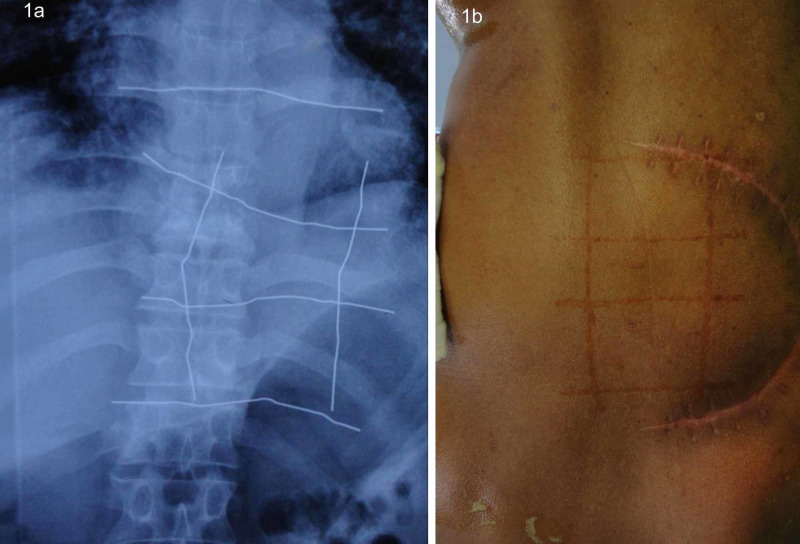
(a) Radiograph showing grids marked with stainless steel wires for anterolateral decompression surgery. (b) Radiograph showing surgical site marked with henna in revision anterolateral decompression.

**Figure 2:**
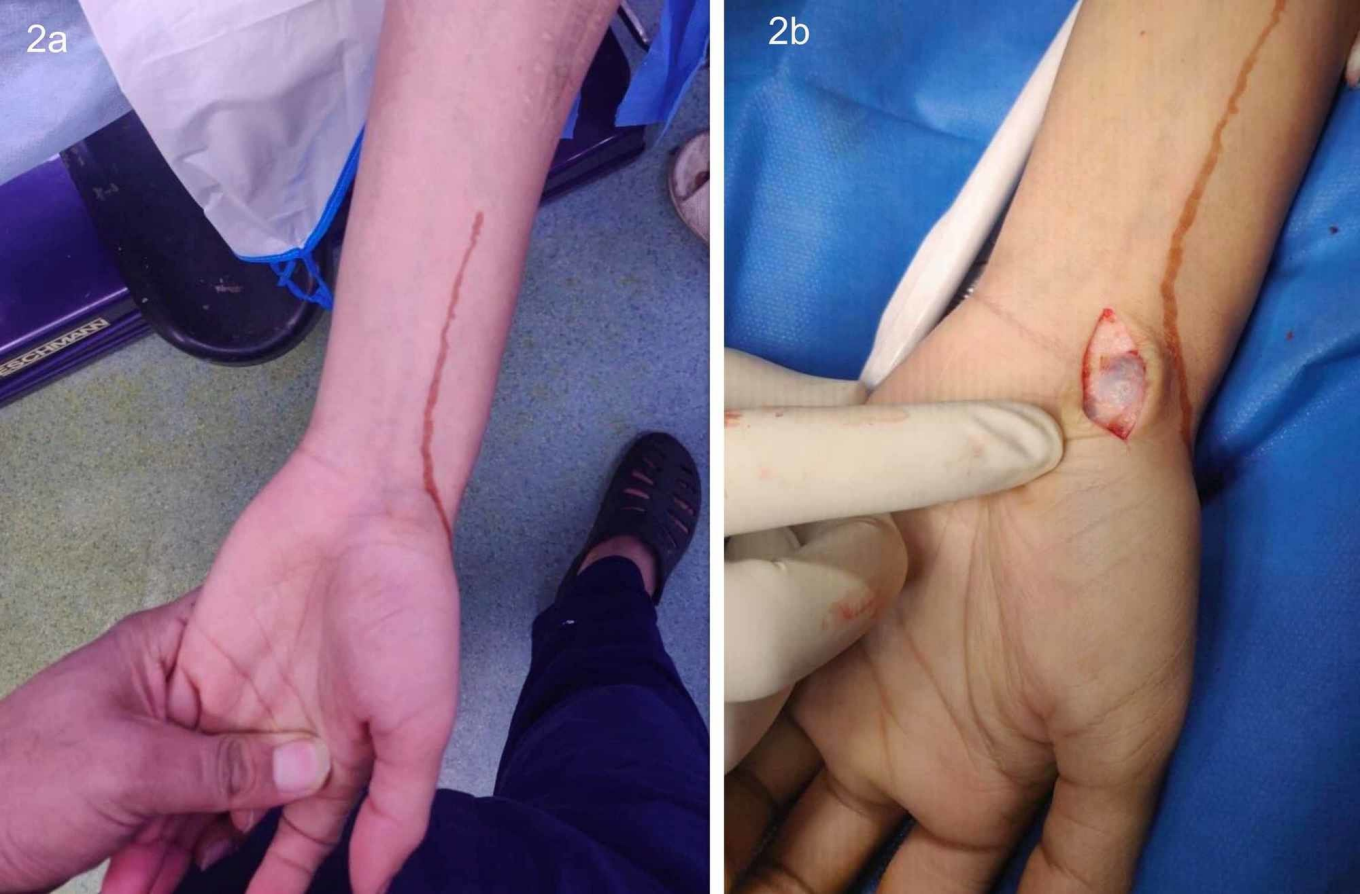
(a): Preoperative clinical photograph showing the radial artery marked with henna. (b) Intraoperative photograph of volar ganglion excision.

A total of 120 patients with varied indications for pre-operative skin marking were enrolled in this study from June 2016 to June 2017 at a tertiary care teaching and referral hospital. Written informed consent was taken from all the patients, and ethical clearance was taken from the institutional committee. All patients were randomly distributed into two groups based on a method similar to picking draw of lots. Every patient was asked to pick a blindfolded slip from a box containing 60 slips each, marked as either henna or permanent marker. Group A patients were those in whom henna was to be used as a marker, and Group B patients were the ones in whom a permanent marker was to be used in marking. We described the technique for application of henna, where the area of interest was first marked by putting flexible stainless-steel wire on the skin and using adhesive bandage to hold the wire, and then plain X-ray films were taken. The wire was carefully removed, and the site of placement of the wire was then marked with henna paste dispensed through a small hole (2-3 mm in diameter) in a paper cone (Figures 1a, 1b). In cases where the blood vessel had to be marked, marking was done during color Doppler ultrasonography where henna paste applied directly over the film of gel over the skin area indicated by the radiologist (Figures 2a, 2b). The henna was allowed to dry for at least two hours and then washed off. Orange to dark brown color was expected at the marking site corroborating with the marks on the X-ray or color Doppler ultrasonography.

All patients were instructed to have a routine pre-operative bath with soap for ensuring good hygiene day before the surgery, and vigorous skin preparation was done using chlorhexidine and ethanol-based scrubs just before surgery in the operating room. For assessment of the efficacy of the two marking methods, the following variables were employed: (1) photographic record: a photograph of the marking site was taken on the evening before the day of surgery, early morning of surgery, and after the routine pre-operative skin preparation with chlorhexidine and ethanol-based scrubs, and saved for comparison purposes; (2) a record was made regarding the successful execution of the intended procedure or if at all an image intensifier had to be used for salvage intra-operatively; (3) any allergic reaction or infection attributable to marking material was noted; (4) all patients were questioned regarding the acceptability of the mark made on their skin and their statements recorded; (5) the final time of the disappearance of the mark was also noted and patients were followed up at 2 weeks, 4 weeks, 8 weeks, and 12 weeks. Chi-square test was used for statistical analysis, and a p-value of <0.05 was considered significant.

## Results

We have tabulated the results in Table 1 for a comprehensive evaluation. Of the 60 markings with henna, only a single case (1.67 %) of fading was observed in a tumor patient where blood vessel course had been marked on evaluating all of the three photographic records for each mark. However, for the permanent marker, in three (5 %) patients, fading of the mark was observed on the morning of surgery after the patients had their routine bath with soap and water, and as many as 10 (16.67%) patients had a mark gone almost out of use after routine pre-operative skin scrubbing. In terms of successful execution of the desired procedure, all but two (3.33%) patients in whom henna was used documented a successful outcome, but as many as 10 (16.67%) patients in whom permanent marker had been used landed up with exposure to intra-operative fluoroscopy. Four out of them were those where vertebral level had been marked for spine surgery. Fading of the mark after vigorous pre-operative skin preparation and the use of fluoroscopy during surgery was significantly low in group A (p<0.05). The median follow-up was 46 days (range: 35-85 days). There was, however, no serious rash or allergy or any wound infection attributable to the marker in any case. Although one patient in whom marking with henna was done did develop a minor rash, it was difficult to attribute the rash to the marker. Marks made with henna disappeared on an average 18 days after the procedure, whereas marks with permanent marker disappeared on an average of nine days after the surgery. Also, the henna application was universally accepted by all the patients, whereas seven patients felt that the permanent marker was unacceptable to them either on religious or cosmetic grounds.

**Table 1:** Showing the results of comparison of henna and permanent skin marker and various indications for use ALD, adjacent level disease

Results	Number of patients in each group		Cases with fading of mark on the morning of surgery		Cases with fading of the mark after scrubbing		No. of cases where fluoroscopy was needed		Allergy/infection	
Indications for use	Group A (henna used)	Group B (permanent marker used)	Group A	Group B	Group A	Group B	Group A	Group B	Group A	Group B
Foreign body removal	11	9	0	1	0	2	1	3	0	0
Dynamization of nail	14	9	0	0	0	1	0	1	0	0
Blood vessel marking in tumor	7	6	0	0	1	1	0	0	0	0
Blood vessel marking in volar ganglion cysts	5	3	0	0	0	1	0	0	0	0
Spine surgery (ALD)	7	8	0	1	0	3	0	4	1 rash	0
Tibial pin insertion in children	8	19	0	0	0	1	0	0	0	0
Sequestrectomy	8	6	0	1	0	1	1	2	0	0
Total cases	60	60	0	3	1	10	2	10	1	0

## Discussion

Henna has been used since antiquity to dye skin, hair, and fingernails. It is also used to dye fabrics, which include silk, wool, and leather [3]. The name henna actually refers to a dye prepared from the flowering plant *Lawsonia inermis*, also known as the henna tree [4]. Henna paste is prepared by mixing crushed leaves and twigs of the henna plant with hot water. When this paste is applied on the skin and left for a few hours, it leaves orange to dark brown stain in the skin, which fades away in 14-21 days [4-5]. Commercially packaged henna intended for use is available in many countries including India. Henna is used during birth and marriage celebrations for ritual skin painting called “Mehndi”. In western countries, people have adopted henna for making temporary tattoos and for organic hair coloring [5].

The use of henna has already been described in breast surgery for locating breast lesion [1], in general surgery for marking perforators and varicosities in chronic venous diseases or stoma sites in urinary or fecal diversions [3], and in radiotherapy [2] where the beam needs to be targeted to a well-defined pathological tissue. For many surgical procedures, even in orthopedics, skin marking is required well before surgery and induction of anesthesia. We would hereby like to highlight some of the important applications we could attribute to henna and discuss the relevant details for each.

In several situations, henna offers a durable, cost-effective readily available alternative to permanent skin markers. May it be dynamization of nail or removal of biologically placed implants, marking the targeted screws with henna reduced intra-operative fluoroscopic exposure to a great extent. It is much easier to locate a sequestrum in a non-discharging osteomyelitis patient if it has been marked on the skin before surgery with henna. For procedures taken up in minor operation rooms, such as the insertion of a tibial pin for skeletal traction in children, marking with henna ensures that there is no iatrogenic violation of the physis. In adults, marking the radial artery for removal of volar ganglion cysts (Figures 2a, 2b) helps avoid iatrogenic injury to the artery.

Additionally, there are several situations where marking with henna offers substantial advantages over the conventionally used permanent skin markers. When ultrasonography is used to locate a translucent foreign body such as a glass piece not visible on a plain X-ray film, the henna paste can be directly applied on the limb over the film of gel ensuring minimal error, which may not be possible with permanent skin markers since it frequently requires a dry surface. In the case of bone tumors that have considerably distorted the normal anatomy, marking the vascular course pre-operatively may prevent a major vascular catastrophe. The localization of the blood vessels, as well as the margins of the tumor, can be done under ultrasound guidance and the same can be marked with henna paste applied directly over the gel film on the limb, thereby ensuring great accuracy. The wide area of application seems to be no problem if henna is to be used considering good cosmetic and mental acceptance of this highly cost-effective stain.

For spine surgery, the marking technique with henna that we used needs to be mentioned distinctly. The common practice in spine surgery is to mark tips of spinous processes with skin markers. However, it is not unusual in anterolateral decompression to perform costotransversectomy at wrong levels as an intra-operatively identifying corresponding rib in the context to the marked spinous process often is difficult. In our study, ribs to be removed were palpated, and a metallic grid made from stainless steel wires was placed along the respective side. After the radiograph was taken, the grid was replaced by marking with henna carefully. Now, the ribs to be removed were identified by locating the respective marked box on the patient in which they were placed against the radiograph, thereby ensuring a zero-error technique (Figures 1a, 1b). However, we would like to caution here that the marking and radiography should preferably be done with the patient in the same position as planned for surgery. Henna merits a special credit here based on its greater patient acceptability and cost-effective property because the area of application is wide and it can withstand a more vigorous pre-operative skin scrubbing as is generally required for spinal procedures. Almost four of our patients needed intra-operative fluoroscopy where marking had been done with a permanent marker in a conventional way for spinal surgery, owing to the lapses in the technique and the fact that the marker was unable to withstand the vigorous pre-operative skin preparation.

Another must mention is wrong-side surgery in the wrong patient. Almost 75 cases are reported of wrong-site surgery in the United States each year [3]. The statistics for the developing world are not there, but it can be expected to be much the same. Many patients are there such as those with carpal tunnel release in whom it is impossible to differentiate the normal and the affected side once the patient is anesthetized. Marking large segments of limbs with this cosmetically acceptable cost-effective durable marker ensures that such catastrophes are minimized.

In three of our patients, surgery was postponed for a week due to similar reasons, but no repeat marking was required before the final procedure in any. The fact that proper pre-operative marking can substitute to a great extent the use of image intensifiers further makes this low-cost skin marker just the ideal alternative for a developing set-up like India. The major problems we faced were mainly in patients who had very lax and mobile skin, where pre-operative skin marking itself is a challenge whatever marking method is used.

## Conclusions

We would conclude that henna is a considerably safe, readily available, cost-effective, non-allergic, non-toxic alternative to a permanent skin marker. Its long-lasting durable nature ensures that even if surgery gets postponed considering anesthetic problems, the surgeon remains unbothered regarding repeat marking or instructing the patient for altering routine toileting or bathing activities. Further studies may be needed to evaluate the safety and patient's acceptability for henna as a skin marker.
